# Acute effects of ambient PM_2.5_ on lung function among schoolchildren

**DOI:** 10.1038/s41598-020-61003-4

**Published:** 2020-03-04

**Authors:** Dandan Xu, Yuan Chen, Lizhi Wu, Shengliang He, Peiwei Xu, Yongli Zhang, Jinbin Luo, Xialiang Ye, Zhijian Chen, Xiaofeng Wang, Xiaoming Lou

**Affiliations:** 1 Zhejiang Provincial Center for Disease Control and Prevention, Hangzhou, Zhejiang Province China; 2 Zhoushan Center for Disease Control and Prevention, Zhoushan, Zhejiang Province China; 3 Jinhua Center for Disease Control and Prevention, Jinhua, Zhejiang Province China; 4 Lishui Center for Disease Control and Prevention, Lishui, Zhejiang Province China

**Keywords:** Environmental impact, Risk factors

## Abstract

Previous studies have found that fine particulate matter (PM_2.5_) air pollution is associated with decreased lung function. However, most current research focuses on children with asthma, leading to small sample sizes and limited generalization of results. The current study aimed to measure the short-term and lag effects of PM_2.5_ among school-aged children using repeated measurements of lung function.This prospective panel study included 848 schoolchildren in Zhejiang Province, China. Each year from 2014–2017, two lung function tests were conducted from November 15^th^ to December 31^st^. Daily air pollution data were derived from the monitoring stations nearest to the schools. A mixed-effects regression model was used to investigate the relationship between PM_2.5_ and lung function. The effect of PM_2.5_ on lung function reached its greatest at 1-day moving average PM_2.5_ exposure. For every 10 μg/m^3^ increase in the 1-day moving average PM_2.5_ concentration, Forced Vital Capacity (FVC) of children decreased by 33.74 mL (95% CI: 22.52, 44.96), 1-s Forced Expiratory Volume (FEV_1_) decreased by 32.56 mL (95% CI: 21.41, 43.70), and Peak Expiratory Flow (PEF) decreased by 67.45 mL/s (95% CI: 45.64, 89.25). Stronger associations were found in children living in homes with smokers. Short-term exposure to PM_2.5_ was associated with reductions in schoolchildren’s lung function. This finding indicates that short-term exposure to PM_2.5_ is harmful to children’s respiratory health, and appropriate protective measures should be taken to reduce the adverse effects of air pollution on children’s health.

## Introduction

Air pollution is a major global concern and causes a variety of health problems, such as increased mortality, hospitalization, and outpatient visits, especially for cardiovascular and respiratory diseases^1–4^. Fine particulate matter (PM_2.5_) is one of the main components of air pollution. Because children are at a crucial stage of growth and development, they are particularly vulnerable to air pollution^4,5^. The adverse effects of PM_2.5_ air pollution on children’s health have been reported in many studies^6–8^, with lung function serving as an effective indicator of early lung disease^9^. By analyzing the relationship between PM_2.5_ and lung function in children, we can better understand the effects of PM_2.5_ air pollution on children’s respiratory health.

Previous studies have found an association between PM_2.5_ air pollution and decreased lung function^10–13^. However, most researchers have focused on children with asthma, creating a small sample size and limiting the extrapolation of results^10,12,14^. In addition, these studies primarily have been carried out in developed countries with low levels of air pollution; developing countries with high levels of air pollution have been comparatively less studied. Another limitation of the current body of literature rests on the fact that most studies have focused on a single lung function indicator, not on multiple lung function indicators simultaneously^11,14,15^. Finally, it must be noted that lung function is affected by various factors, such as age, gender, and disease state; however, the modification effects of these factors on the relationship between PM_2.5_ and lung function remain ambiguous^10–12^. Due to these shortcomings, it is possible to state that the impact of PM_2.5_ pollution on lung function in children has yet to be fully and accurately understood.

This study sought to explore the short-term and lag effects of PM_2.5_ among school-aged children using repeated measurements of lung function including Forced Vital Capacity (FVC), 1-s Forced Expiratory Volume (FEV_1_), and Peak Expiratory Flow (PEF). We also considering the modification effects of other factors. These results can serve as a scientific basis demonstrating the harm of PM_2.5_ to children’s respiratory health, creating a catalyst for protecting children’s health.

## Methods

### Study population and design

This prospective panel study was conducted in Zhejiang Province, located in eastern China. Three cities were chosen for investigation: Jinhua, Lishui, and Zhoushan. Jinhua is located in the middle of Zhejiang Province and is hill-and-basin in landform. Lishui is located in the southwest of Zhejiang Province and is mainly in mountainous and hilly terrain. Zhoushan is an island city located in the northeast of Zhejiang Province. These three cities have good representation in Zhejiang Province. We selected 1–2 primary schools within 2 km of national air pollution monitoring stations before recruiting primary school students in grades 3–5 to fill in questionnaires and undergo lung function tests. In each school, 50 students were randomly selected from each grade, and a total of 150 students were surveyed each year. The questionnaires were completed by students and their parents. From 2014–2017, we conducted questionnaire surveys and lung function tests from November 15^th^ to December 31^st^ each year, during which time the particulate matter air pollution was serious. Table S1 indicates the number of participants and the number of lung function measurements in each city. Each year we recruited new students in grade 3, and students who progress to grade 6 left the study. So some students measured their lung function 6 times, while other students only measured it once or twice. We measured lung function on a total of 1,245 individuals one to six times and collected 3,418 lung function measurements. Because the lung function data involves repeated measurement, we used a linear mixed-effects regression model for data analysis and extracted data with more than 2 lung function measurements. Following this step, data analysis included 848 students with a total of 3,021 lung function measurements.

### Ethics statement

The study was approved by the Medical Ethics Committee of the Zhejiang Provincial Center for Disease Control and Prevention. The research was conducted in accordance with the Declaration of Helsinki. Participants enrolled in this study and their parents gave written informed consent for participation.

### Data collection

#### Lung function measurement

Lung function tests were performed in schools, and the rooms used for testing were relatively independent, quiet, bright, clean, and temperature-friendly. Tests were conducted by local medical staff who are responsible for lung function measurement in hospitals or physical examination centers using the same electronic spirometer (SPirOAnalyzer ST-75, FUKUDA SANGYO, Japan). From November 15^th^ to December 31^st^ each year, lung function was measured twice in Jinhua and Lishui, once in November and once in December; in Zhoushan, lung function was measured once during this period each year, because Zhoushan is an island city, less air pollution. All lung function tests were conducted in the morning and according to published standards^16^. Before measuring lung function, the electronic spirometer was calibrated according to changes in temperature, humidity, and atmospheric pressure. Then, the children’s parameters were put into the electronic spirometer, including age, sex, height, and weight. With the participant standing and wearing a nose clip, pulmonary function tests were repeated in order to obtain at least three reproducible forced expiratory flow curves. Lung function indicators included FVC, FEV_1_, and PEF.

#### Air pollution data

**Figure 1:**
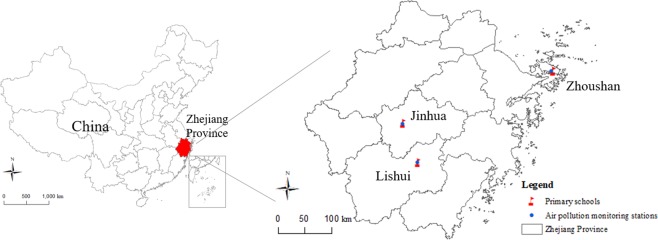
Map of the study area, including the location of the air pollution monitoring stations and the primary schools. The map was created using the ArcGIS software (version 10.2, 64-bit).

Daily air pollution data from January 1, 2014 to December 31, 2017 were derived from the fixed-site monitoring stations of the National Air Pollution Monitoring System. We selected the monitoring stations nearest to the schools (Fig. 1). Air pollution indicators included PM_2.5_, sulfur dioxide (SO_2_), nitrogen dioxide (NO_2_), carbon monoxide (CO), and daily 8-h maximum ozone (O_3–8h_).

#### Covariates

Participants’ basic information, including family situation, lifestyle, living environment, and disease history, was obtained via questionnaires completed by the students and their parents. To control for the influence of meteorological factors on lung function, we also obtained data regarding the daily mean temperature and relative humidity from January 1, 2014 to December 31, 2017. We collected this information for each city from China’s Meteorological Administration’s data network. Our analysis included the following covariates: age (continuous), sex (boys, girls), height (continuous), weight (continuous), passive smoking status (yes, no), asthma (yes, no, we determined asthma status through question “Have you been diagnosed with asthma by a doctor?” in the questionnaire.), father’s education level (primary school and below, junior high or high school, college and above), whether the family uses natural gas for cooking (yes, no), whether the family uses air purifiers at home (yes, no), temperature (continuous), and relative humidity (continuous).

### Statistical analysis

We used a mixed-effects regression model with study participants and districts as random effects in order to investigate the relationship between PM_2.5_ and lung function^17^. The effect estimates were expressed as the increase in lung function per 10-μg/m^3^ increase in PM_2.5_ concentration. The mixed-effects model was constructed using Eq. (1).

1 $${{\rm{Y}}}_{ijk}={\beta }_{0}+{\beta }_{1}{{\rm{PM}}}_{2.5}+{\beta }_{2}{X}_{1ijk}+\cdots +{\beta }_{{\rm{n}}}{X}_{nijk}+{\xi }_{j}+{\xi }_{k}+{e}_{ijk};$$

where Y_ijk_ represents the lung function indicators (FVC, FEV_1_, PEF); β_0_ is the overall intercept; β_1_ is the regression coefficient for PM_2.5_; β_2_ …β_n_ are the regression coefficients for the covariates in the model; ξ_j_ is the random effect for the study participant; ξ_k_ is the random effect for the study district; j represents the study participant; k represents the study district; i identifies the workday; and e_ijk_ is the residual error term.

Using the model, we measured exposure to PM_2.5_ concentration at the current day (lag0) and at period lags (lag01, lag02, lag03, lag04, lag05). For example, lag01 would be the 1-day moving average PM_2.5_ exposure (the average PM_2.5_ concentration of the current day and 1day before lung function measurement). Two-pollutant models were used to adjust for the potential confounding effects of co-pollutants. After correlation analysis, CO or O_3–8h_ was adjusted for in the two-pollutant models.

We also performed stratified analyses by sex (boys, girls), smoking at the child’s home (yes, no), asthma (yes, no), whether the family uses natural gas for cooking (yes, no), and whether the family uses air purifiers at home (yes, no) in order to explore the association between 1-day moving average PM_2.5_ exposure and lung function indicators.

The following statistical formula was used to calculate whether or not the differences between groups were statistically significant (P < 0.05, Eq. 2).

2 $$({b}_{1}-{b}_{2})\pm 1.96\times \sqrt{S{E}_{1}^{2}+S{E}_{2}^{2}};$$

Where b_1_ and b_2_ are the effect estimates of the two groups, and SE_1_ and SE_2_ are the standard errors of the two effect estimates, respectively.

Sensitivity analyses were performed to test the robustness of the results. We added a confounding factor (whether the participant’s home had pets) in the analysis model. In addition, we restricted data analysis to participants who did not have asthma and who did not use an air purifier at home.

All statistical tests were two-sided, and P < 0.05 was considered statistically significant. Data analysis was performed using R version 3.5.1 software and the lme4 package.

## Results

### Characteristics of study participants

Table 1 illustrates the characteristics of the study population. A total of 848 participants and 3,021 lung function measurements were included in data analysis. The average age of the students was 9.74 years, with boys and girls accounting for 53.30% and 46.70% respectively. The percentage of children exposed to second-hand smoke was high at 59.28%. Households using natural gas accounted for 14.98%, and a few households used air purifiers, accounting for only 9.91%. There were very few students with asthma, only 2.12%.

**Table 1:** Characteristics of the study population.

Variables	n/N	Mean ± SD or Percent
Age		9.74 ± 0.99
Sex		
Boys	452/848	53.30
Girls	396/848	46.70
Height		141.05 ± 9.09
Weight		34.53 ± 8.93
Father’s education		
Primary school and below	69/848	8.14
Junior high or high school	523/848	61.67
College and above	256/848	30.19
Passive smoking	444/749	59.28
Use of natural gas for cooking	127/848	14.98
Purifier	84/848	9.91
Asthma	18/848	2.12
Pet	109/848	12.85

### Results of descriptive data analysis and correlation analysis

The results of our descriptive analysis, including lung function, daily air pollution, and daily meteorological data were shown in Table 2. The table presents the means, standard deviations, minimums, quartiles, medians, and maximum measurements for lung function indicators, air pollution data, and meteorological data. The PM_2.5_ concentration was 67.58 ± 41.76 μg/m^3^, it is much higher than the World Health Organization’s ambient air quality guidelines for 24-h average PM_2.5_ of 25 mg/m^3^, but lower than the Chinese ambient air quality guidelines of 75 mg/m^3^. The minimum and maximum concentration was 9.00 μg/m^3^ and 156.00 μg/m^3^, respectively. Descriptive statistics on air pollution data for each city are shown in Table S2.

**Table 2:** Descriptive statistics for lung function indicators, daily air pollution, and daily meteorological data.

Variables	Mean	SD	Min	P25	P50	P75	Max
**Lung function**							
FVC, L	2.65	1.26	0.57	1.72	2.26	3.36	7.48
FEV_1_, L	2.46	1.26	0.27	1.55	2.04	3.17	7.17
PEF, L/s	4.66	2.43	0.54	2.79	4.01	6.28	9.99
**Air pollution data**							
PM_2.5_, µg/m^3^	67.58	41.76	9.00	36.00	62.00	98.00	156.00
SO_2_, µg/m^3^	14.82	9.07	3.00	9.00	12.00	19.00	37.00
NO_2_, µg/m^3^	42.23	19.25	16.00	28.00	38.00	48.00	85.00
CO, mg/m^3^	0.95	0.27	0.60	0.70	0.99	1.00	1.43
O_3_8h_, µg/m^3^	80.01	57.74	4.00	50.00	63.00	103.00	215.00
**Meteorological data**							
Temperature, °C	15.22	6.07	3.40	10.00	15.40	20.50	25.20
RH, %	77.48	11.06	54.00	72.00	77.60	84.00	96.00

Table S3 shows the correlation coefficients between daily mean measures of outdoor air pollution, temperature, and relative humidity. There were high positive correlations between PM_2.5_, SO_2,_ and NO_2_, and the correlation coefficient between PM_2.5_ and SO_2_ was the largest at 0.85 (P < 0.001). O_3–8h_ was negatively correlated with these pollutants. The correlation coefficient between temperature, relative humidity, and these air pollutants was small (P > 0.05).

### Effects of PM2.5 on lung function

**Figure 2:**
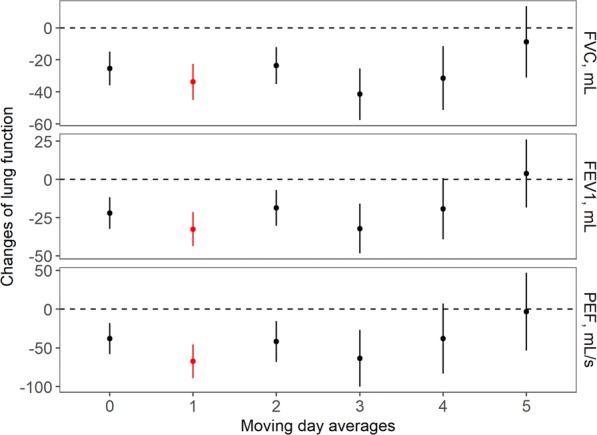
Effects of current day and moving average PM_2.5_ exposure on lung function. These are estimates from the mixed-effect regression models, with adjustments for age, sex, height, weight, passive smoking status, asthma, father’s education level, whether the family uses natural gas for cooking, whether the family uses air purifiers at home, temperature, and relative humidity.

Figure 2 shows the changes in lung function per 10 μg/m^3^ increase in PM_2.5_ concentrations on the current day as well as the moving average exposure. A 10 μg/m^3^ increase in the current day PM_2.5_ concentration corresponded to a decrease in 25.45 mL (95% CI: 14.94, 35.96) FVC, 22.01 mL (95% CI: 11.55, 32.47) FEV_1_, and 37.96 mL/s (95% CI: 17.80, 58.13) PEF. When exposed to the 1-day moving average PM_2.5_ concentration before lung function measurement, children demonstrated the largest changes in lung function, with a 33.74 mL (95% CI: 22.52, 44.96) decrease in FVC, a 32.56 mL (95% CI: 21.41, 43.70) decrease in FEV_1_, and a 67.45 mL/s (95% CI: 45.64, 89.25) decrease in PEF.

### Results of two-pollutant models

Table 3 shows the results of the two-pollutant modes. The correlation coefficients between PM_2.5_ and SO_2_, and between PM_2.5_ and NO_2_ were very high, which may indicate problems of multi-colinearity. As a result, we only included CO and O_3–8h_ in the two-pollutant model for data analyses. In two-pollutant models with CO or O_3–8h_, the PM_2.5_ effect remained significant (P < 0.05), and the effect values changed little when compared to those of the single-pollutant model.

**Table 3:** The results of two-pollutant models with CO or O_3–8h_.

Pollutants	FVC (mL)	FEV_1_ (mL)	PEF (mL/s)
	Estimate (95% CI)	Estimate (95% CI)	Estimate (95% CI)
PM_2.5_	−33.74 (−44.96,−22.52)	−32.56 (−43.7,−21.41)	−67.45 (−89.25,−45.64)
+CO	−43.77 (−58.63,−28.91)*	−42.16 (−56.92,−27.39)*	−71.58 (−100.37,−42.79)*
+O_3–8h_	−31.55 (−43.37,−19.73)*	−28.63 (−40.38,−16.88)*	−65.41 (−88.34,−42.49)*

*These estimates are from the two-pollutant regression models, with adjustments for age, sex, height, weight, passive smoking status, asthma, father’s education level, whether the family uses natural gas for cooking, whether the family uses air purifiers at home, temperature, and relative humidity.

### Results of stratified analyses

**Figure 3:**
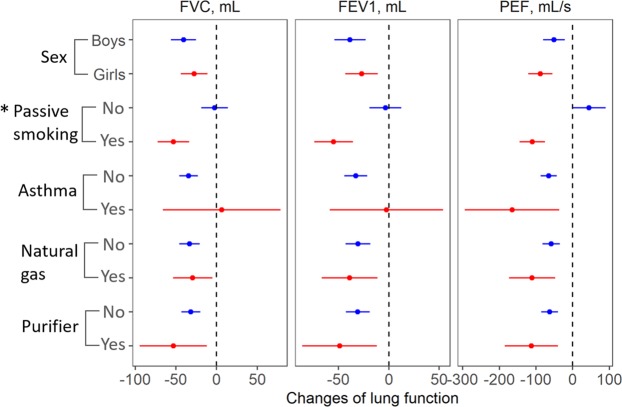
The results of stratified analyses for the association between 1-day moving average PM_2.5_ exposure and lung function indicators. *Indicates that the difference between groups was statistically significant.

Figure 3 presents the results of stratified analyses. The results stratified by sex revealed that PM_2.5_ more strongly affected FVC and FEV_1_ for boys than for girls. The effect on PEF was weaker on boys than girls, but these differences were not statistically significant. When stratified by passive smoking status, no significant PM_2.5_ effects were observed for the non-passive smoking group. However, for children who are exposed to second-hand smoke, there was a reduction of 53.12 mL (95% CI: 33.76, 72.49) in FVC, a reduction of 54.80 mL (95% CI: 35.61, 73.98) in FEV_1_, and a reduction of 110.12 mL/s (95% CI: 75.36, 144.89) in PEF for each increase of 10 μg/m^3^ in the 1-day moving average PM_2.5_ concentration. The differences between the two groups (exposed to passive smoking and not) were statistically significant (P < 0.05). When stratified by asthma status, no significant PM_2.5_ effects were observed for FVC and FEV_1_ in the group of asthmatics, but there was a greater effect for PEF compared to the group of non-asthmatics. When we stratified based on the use of natural gas and air purifiers, we found no significant differences between these subgroups in terms of PM_2.5_ effects (P > 0.05).

### Results of sensitivity analyses

The results of sensitivity analyses were shown in Table S4. When we added the confounding factor (whether the participant’s home had pets) into the analysis model, the effect value of PM_2.5_ on lung function was similar to the result found in the primary model analysis. When restricting data analysis to participants who did not have asthma and who did not use an air purifier at home, the effect value also changed little compared to the main model. These results indicate stability in our main model.

## Discussion

Based on repeated lung function measurements among primary school students in Zhejiang, we found that short-term exposure to PM_2.5_ was associated with reductions in lung function indicators. In addition, the effects of PM_2.5_ on lung function had significant lag effects, with the largest effect occurring on the 1-day moving average PM_2.5_ exposure. Based on the stratified analyses, our results indicate that children exposed to second-hand smoke were more sensitive to the effects of PM_2.5_. Sex, asthma status, the use of natural gas, and air purifiers did not affect the relationship between PM_2.5_ and lung function.

Our study found that an increase in the current day PM_2.5_ concentration was associated with a statistically significant decrease in lung function indicators, indicating that PM_2.5_ can affect children’s lung function in a short amount of time. Similar findings have been found in previous studies^10,11,14,18^. A panel study with 309 children conducted in Brazil found that, for an increase of 10 mg/m^3^ in same-day PM_2.5_ exposure, there was an average reduction of 0.26 l/min (95% CI: 0.49, 0.04) in the PEF^11^. Another Japanese study found that increased 24-hour mean concentrations of PM_2.5_ were associated with a decrease in both morning and evening PEF (−3.0 l/minute; 95% CI: −4.6, −1.4 and −4.4 l/minute; 95% CI: −7.1, −1.7, respectively)^14^. In China, there exist few studies regarding the effects of short-term particulate matter exposure on children’s lung function. A panel study comprised of 37 healthy college students in Wuhan, China found that a 10 μg/m^3^ increase of outdoor PM2.5 was associated with a decrease of 1.54 L/min in evening PEF (95% CI: −3.03,−0.04) L/min^15^. Many studies have focused on a single indicator of lung function, including the three aforementioned studies, which focused only on PEF. Our research, however, included the FVC, FEV_1,_ and PEF—three indicators of lung function. After unit conversion, we note that our PEF effect value was similar to those reported in studies from Wuhan and Japan. However, our results differ greatly from those from Brazil, which may be due to different climates, latitudes, and participant characteristics.

We also found that the effect of PM_2.5_ on lung function reached its greatest at 1-day moving average exposure., and the effect of PM_2.5_ exposure with 3-day moving average on lung function remained statistically significant. This finding indicates that there is a significant lag effect of PM_2.5_ on lung function. When an air pollution incident occurs, people’s symptoms may persist for several days, and we need to pay close attention to health protection at this time. Previous studies also have found a lag effect of PM_2.5_ on lung function, with a lag time of 1–5 days^11–13^. Jacobson *et al*. found an average reduction of 0.37 l/min (95% CI: 0.68, 0.07) in the PEF for lag0–1 and an average reduction of 0.38 l/min (95% CI: 0.71, 0.04) for lag0–2 cumulative exposure per increase of 10 mg/m^3^ in PM_2.5_^11^. A study in the United States found robust associations between PM and FEV_1_ for lag0 and 2-, 4-, and 5-day averages^12^. A study of 1,494 non-asthmatic children in Taiwan showed a negative association between the lag1 day exposure of particulate matter and FVC, FEV_1_, and maximum mid-expiratory flow (MMEF). The lag2 day demonstrated less significant effects on lung function than lag1 day^13^, results that mirror the current study.

In our two-pollutant models with CO or O_3–8h_, the PM_2.5_ effect remained statistically significant (p < 0.05), meaning that PM_2.5_ demonstrated a robust effect on lung function. Previous studies have demonstrated similar results^10,13,15^. These studies all showed that the association between PM_2.5_ and lung function persisted in two-pollutant models with O_3_, NO_2,_ and SO_2_. In our study, the correlations between PM_2.5_ and SO_2_, and between PM_2.5_ and NO_2_ were very high and there may be multi-colinearity; as such, we only adjusted CO or O_3–8h_ in the two-pollutant models.

In our study, we found that children exposed to second-hand smoke were more sensitive to the effect of PM_2.5_ on lung function. Sex, asthma status, the use of natural gas, and the use of air purifiers did not affect the relationship between PM_2.5_ and lung function. Our results suggest that second-hand smoke increases the vulnerability of children to air pollution, severely affecting children’s lung function. Regarding the modification effects of these factors, previous studies have provided mixed results. For the sex comparison, a Canadian study reported similar findings to ours, noting no significant differences between genders. A study in Shenyang, China also reported similar findings^19^. However, research from southern California found significantly stronger associations between FEV_1_ and PM in boys than in girls. Inferences from this study remain somewhat limited because the researchers compared responses of five girls to those of 14 boys^12^. For asthma’s modification effect, Jacobson *et al*. found a reduction in PEF ranging from 0.38 l/min (95% CI: 0.63, 0.13) to 0.53 l/min (95% CI: 0.90, 0.16) for each increase of 10 mg/m^3^ in PM_2.5_ in the subgroup of non-asthmatic children. However, the researchers found no significant effect in the group of asthmatic children when analyzed separately. They offered a possible explanation for these results by noting that asthmatic subjects may take medication when air quality deteriorates^11^. A study conducted in Seattle reported adverse effects from centrally-monitored PM_2.5_ on the FEV_1_ of children with asthma, but only if they were not receiving anti-inflammatory medications^20^. Therefore, our results also may reflect the use of drugs in children with asthma, leading to no apparent effects of PM_2.5_ exposure on asthmatic lung function. Unfortunately, we did not collect information regarding students’ use of medications. Few studies have focused on the modification effects of passive smoking, the use of natural gas, or the use of air purifiers, making it difficult to compare our findings to previous studies. It is recommended that future studies analyze the modification effects of these factors.

This study has several advantages. First, our panel study recruited a large sample of 848 students and collected 3,021 lung function measurements. The large number of lung function measurements increased the power of our statistical analysis, rendering our results more stable. Second, we used multiple lung function indicators to investigate the effects of short-term PM_2.5_ exposure on lung function, while previous studies primarily relied on a single lung function indicator^10,11,13,15^. Third, we performed a mixed-effects regression model to analyze repeated measurement data and to control for a variety of possible confounding factors, resulting in a highly reliable analysis of results. Meanwhile, the repeated measurement analysis helped to reduce the impact of individual and other factors that may influence the relationship between PM_2.5_ and lung function. Fourth, our stratified analyses based on several factors assisted in observing the modification effects of these factors on the relationship between PM_2.5_ and lung function. These findings may help to inform us regarding vulnerable populations that may be more susceptible to decreased lung function due to air pollution.

This study also has some unavoidable limitations. First, we used outdoor rather than indoor or personal air pollution data; the data were obtained from the monitoring station nearest each school. In addition, the students’ exposure to air pollution away from school was not measured. However, there exists a school district division policy in China, and students must study near their homes. Therefore, the monitoring stations closest to the schools likely provide sound assessments of both school and home exposure to outdoor air pollution. In addition, Janssen *et al*. reported that the median Pearson correlation coefficient between personal and outdoor concentrations of PM_10_ was 0.71 when not exposed to second-hand smoke, indicating that changes in outdoor levels reflect personal changes^21^. Second, we did not collect information regarding student medications and thus could not analyze the effects of medications on the relationship between PM_2.5_ and lung function. However, we conducted a stratified analysis based on asthma and obtained the effect of PM_2.5_ on the lung function of healthy students. Because there were very few students with asthma (18 students), this omission would have little impact on our findings.

## Conclusions

Based on repeated lung function measurements among primary school students in Zhejiang, we found that short-term exposure to PM_2.5_ was associated with reductions in lung function indicators, with the largest effect occurring on the 1-day moving average PM_2.5_ exposure. In addition, children exposed to second-hand smoke were more sensitive to the effects of PM_2.5_. Our results suggest that short-term exposure to PM_2.5_ is harmful to children’s respiratory health, and appropriate protective measures should be taken to reduce the adverse effects of air pollution on children’s health. Our research thus is of great significance for the control of air pollution and the protection of children.

## Supplementary information

Supplemental material.

## Data Availability

The datasets generated during and/or analysed during the current study are available from the corresponding author on reasonable request.

## References

[1] Samet JM, Dominici F, Curriero FC, Coursac I, Zeger SL (2000). Fine particulate air pollution and mortality in 20 U.S. Cities, 1987–1994. N. Engl. J. Med..

[2] Barnett AG (2006). The effects of air pollution on hospitalizations for cardiovascular disease in elderly people in Australian and New Zealand cities. Env. Health Perspect..

[3] Wang KY, Chau TT (2013). An association between air pollution and daily outpatient visits for respiratory disease in a heavy industry area. PLoS One..

[4] WHO. 2007 (2005). Air quality guidelines: Global update. Particulate matter, ozone, nitrogen dioxide and sulfur dioxide. Indian. J. Med. Res..

[5] Odajima H, Yamazaki S, Nitta H (2008). Decline in peak expiratory flow according to hourly short-term concentration of particulate matter in asthmatic children. Inhal. Toxicol..

[6] Gouveia N, Junger WL, Investigators E (2017). Effects of air pollution on infant and children respiratory mortality in four large Latin-American cities. Env. Pollut..

[7] O’Connor GT (2008). Acute respiratory health effects of air pollution on children with asthma in us inner cities. J. Allergy Clin. Immunol..

[8] Gauderman WJ (2000). Association between air pollution and lung function growth in southern California children. Am. J. Resp. Crit. Care..

[9] Pellegrino R (2005). Interpretative strategies for lung function tests. Eur. Respir. J..

[10] Dales R, Chen L, Frescura AM, Liu L, Villeneuve PJ (2009). Acute effects of outdoor air pollution on forced expiratory volume in 1 s: A panel study of schoolchildren with asthma. Eur. Respir. J..

[11] Jacobson LDSV, Hacon SDS, Castro HAD, Ignotti E, Artaxo P (2012). Association between fine particulate matter and the peak expiratory flow of schoolchildren in the Brazilian subequatorial amazon: A panel study. Env. Research..

[12] Delfino RJ (2004). Association of fev1 in asthmatic children with personal and microenvironmental exposure to airborne particulate matter. Env. Health Perspect..

[13] Chen CH, Chan CC, Chen BY, Cheng TJ, Leon GY (2015). Effects of particulate air pollution and ozone on lung function in non-asthmatic children. Env. Research..

[14] Yamazaki S (2011). Effect of hourly concentration of particulate matter on peak expiratory flow in hospitalized children: A panel study. Env. Health..

[15] Zhang Y (2015). Short-term effects of fine particulate matter and temperature on lung function among healthy college students in Wuhan, China. Inter. J. Env. Res. Pub Heal..

[16] Miller MR (2005). Standardisation of spirometry. Eur. Respir. J..

[17] Liu LC, Hedeker D (2006). A mixed-effects regression model for longitudinal multivariate ordinal data. Biometrics..

[18] Zwozdziak A (2016). Influence of pm1 and pm2.5 on lung function parameters in healthy schoolchildren-a panel study. Env. Sci. Pollut. Res. Int..

[19] Kasamatsu J, Shima M, Yamazaki S, Tamura K, Sun G (2006). Effects of winter air pollution on pulmonary function of school children in Shenyang, China. Int. J. Hyg. Envir Heal..

[20] Trenga CA (2006). Effect of particulate air pollution on lung function in adult and pediatric subjects in a Seattle panel study. Chest..

[21] Janssen NA (1998). Personal sampling of particles in adults: Relation among personal, indoor, and outdoor air concentrations. Am. J. Epidemiol..

