# Supplementary material for: Acute effects of ambient PM2.5 on lung function among schoolchildren
Source: Sci Rep. 2020 Mar 4;10:4061. doi: 10.1038/s41598-020-61003-4 (PMC7055357; doi:10.1038/s41598-020-61003-4)
Supplement: Supplementary file 1 — Supplemental material. [file 41598_2020_61003_MOESM1_ESM.pdf]

Supplemental material

Acute effects of ambient $PM_{2.5}$ on lung function among schoolchildren

Dandan Xu, Yuan Chen, Lizhi Wu, Shengliang He, Peiwei Xu, Yongli Zhang, Jinbin Luo, Xialiang Ye, Zhijian Chen, Xiaofeng Wang, Xiaoming Lou

Table S1. The number of participants and the number of lung function measurements in each city.

**Lung function measurement times**	**Number of people**			
	**Jinhua**	**Lishui**	**Zhoushan**	**Total**
**1**	10	0	387	397
**2**	169	119	106	394
**3**	1	0	2	3
**4**	120	120	0	240
**5**	2	0	0	2
**6**	149	60	0	209
**Total**	451	299	495	1245

Table S2. Descriptive statistics on daily air pollution data for each city.

Variables	Mean ±SD		
	Jinhua	Lishui	Zhoushan
$PM_{2.5}$, μg/m^3	84.03 ±48.00	52.60 ±21.66	35.56 ±17.67
SO2, μg/m^3	19.58 ±9.94	10.05 ±3.35	7.76 ±4.28
NO2, μg/m^3	53.01 ±19.81	31.03 ±8.42	28.13 ±11.97
CO, mg/m^3	0.99 ±0.23	0.93 ±0.30	0.69 ±0.16
O3_8h, μg/m^3	79.44 ±70.90	81.66 ±35.72	76.00 ±16.27

Table S3. Pearson correlation coefficients between daily mean measures of outdoor air pollution, temperature and relative humidity during study period.

	**$PM_{2.5}$**	**SO_2**	**NO_2**	**CO**	**$O_{3\text{-}8h}$**	**Tmean**	**RH**
**$PM_{2.5}$**	1.00	0.85^{**}	0.84^{**}	0.65^{*}	-0.09	-0.23	-0.12
**SO_2**		1.00	0.76^{**}	0.45	-0.09	-0.09	-0.16
**NO_2**			1.00	0.48	-0.29	-0.26	0.05
**CO**				1.00	-0.10	0.09	0.21
**$O_{3\text{-}8h}$**					1.00	0.37	-0.50
**Tmean**						1.00	0.33
**RH**							1.00

$^{**}P<0.001$, $^{*}P<0.05$

Table S4. Results of sensitivity analyses using the adjusted model to evaluate the association between 1-day moving average $PM_{2.5}$ exposure and lung function.

Model	FVC（mL）	FEV1（mL）	PEF（mL/s）
	Estimate (95%CI)	Estimate (95%CI)	Estimate (95%CI)
**Main model**	-33.74(-44.96,-22.52)	-32.56(-43.7,-21.41)	-67.45(-89.25,-45.64)
**Sensitivity model 1**	-33.76(-44.98,-22.53)	-32.61(-43.76,-21.46)	-67.55(-89.37,-45.74)
**Sensitivity model 2**	-32.34(-44.22,-20.45)	-31.05(-43.05,-19.05)	-60.27(-83.63,-36.92)

* Sensitivity model 1: Adding the confounding factor (whether or not the participant's home had pets) into the main analysis model.

Sensitivity model 2: Restricting data analysis to participants who did not have asthma and who did not use an air purifier at home.

Air pollution data

Daily air pollution data from January 1, 2014 to December 31, 2017 were derived from the fixed-site monitoring stations of the National Air Pollution Monitoring System. It is a kind of passive monitoring and continuous sampling. We conducted a quality review of air pollution data and found that there were no abnormal data and duplicate data, and the rate of missing data was also low. During the study period, in three cities, the loss rate of $PM_{2.5}$ was 5.1%, the loss rate of NO_2 was 5.1%, the loss rate of SO_2 was 5.6%, the loss rate of CO was 5.6%, the loss rate of O_3 was 8.9%. We removed the missing data during data analyses.
